# Adhesive properties of Aphrophoridae spittlebug foam

**DOI:** 10.1098/rsif.2023.0521

**Published:** 2024-01-10

**Authors:** Hannelore Hoch, Martin Pingel, Dagmar Voigt, Urs Wyss, Stanislav Gorb

**Affiliations:** ^1^ Museum für Naturkunde, Leibniz-Institut für Evolutions- und Biodiversitätsforschung, Invalidenstraße 43, 10115 Berlin, Germany; ^2^ Botany, Faculty of Biology, Technische Universität Dresden, 01062 Dresden, Germany; ^3^ Entofilm, Dahlmannstraße 2a, 24103 Kiel, Germany; ^4^ Department of Functional Morphology and Biomechanics, Zoological Institute, Christian-Albrechts-Universität zu Kiel, Am Botanischen Garten 1–9, 24098 Kiel, Germany

**Keywords:** cryo-SEM, defence, foam nest, insect cuticle, pull-off force, surfactant

## Abstract

*Aphrophora alni* spittlebug nymphs produce a wet foam from anal excrement fluid, covering and protecting themselves against numerous impacts. Foam fluid contact angles on normal (26°) and silanized glass (37°) suggest that the foam wets various substrates, including plant and arthropod surfaces. The pull-off force depends on the hydration state and is higher the more dry the fluid. Because the foam desiccates as fast as water, predators once captured struggle to free from drying foam, becoming stickier. The present study confirms that adhesion is one of the numerous foam characteristics resulting in multifunctional effects, which promote spittlebugs' survival and render the foam a smart, biocompatible material of biological, biomimetic and biomedical interest. The sustainable ‘reuse' of large amounts of excrement for foam production and protection of the thin nymph integument suggests energetic and evolutionary advantages. Probably, that is why foam nests have evolved in different groups of organisms, such as spittlebugs, frogs and fish.

## 1. Introduction

Fluidic foams in nature have fascinated researchers for centuries but are not fully understood. They occur in the sea [1–3], horse sweat [4] and foam nests are known from subtropical and tropical tree frog eggs [5–12], fish eggs [13,14], as well as from spittlebug nymphs [e.g. 15–18]. The latter, also known as cuckoo spittle, is released by juvenile instars of froghoppers (Hemiptera, Cercopoidea) belonging to two families (Aphrophoridae, Cercopidae), with a total of more than 2220 species worldwide [19]. The presumably energy-intensive production of foam nests results from the synergistic coaction of morphology, physiology and behaviour [20]. The Aphrophoridae spittlebugs attach to plants (e.g. stems, leaves) with their bodies upside down and feed on a wide host plant variety by inserting the stylets of their mouthparts into the main transpiration stream of plant xylem tissue [16,18,21,22]. Because xylem sap contains more than 95% water and only a low portion of organic matter having 98% amino acids [23–25], the insects must take up large amounts of xylem sap but retain very low proportions of ions [26,27]. Only 33% of the ingested energy is assimilated by spittlebug nymphs [28]. Most xylem sap passes the bugs' alimentary canal before being emitted at the anus as a clear fluid [17,29]. Spittlebugs excrete 150–280 times their body mass fluid every 24 h [30] at a velocity of 50 cm s^−1^ or 30 mg h^−1^ per mg of insect [23,25].

The alkaline fluid (pH 6.8–7.0) contents differ between species; however, overall, comprising 99.4% water, 0.1% organic matter and 0.4% inorganic matter (Mg, Ca, Si, K, Na, Cu, Al, Ti, Fe, S, CO, C, P) [31–34], similar to the host plant juices [35,36]. In addition, the Aphrophoridae foam fluid contains proteinaceous products of a glycoprotein nature and large quantities of a highly negatively charged acid mucopolysaccharide secreted by Malpighian tubules [34,36–43]. Recent biochemical analyses of *Aphrophora alni* nymphs' foam on black alder tree leaves turned out a carbohydrate polymer similar in sugar unit composition to fucoidate isolated from brown seaweed, with the major sugar units fucose (17.0–35.6% of the secretion dry weight), glucose (6.1–16.2%), mannose (1.5–9.9%), rhamnose (0.4–1.3%) and xylose (0.06–0.1%). Also, fatty acids, myo-inositol, pinitol, aliphatic hydrocarbons, waxes and aliphatic derivatives of 2(3*H*)-furanone were detected. The protein content of the analysed secretion did not exceed 0.12% of dry weight, while sugar units reached 92% of dry weight [44]. The content concentration increases with each spittlebug instar [45]. Soon after release, the fluid adheres to plant surfaces and nymphs, surrounding the nymph body and accumulating ventrally. The abdominal tip is temporarily held outside the fluid to take up the air in a ventral hollow tube-shaped channel formed by large tergite plates [17,31,46]. The bugs breathe into the fluid using this air reservoir and develop foam [17,46,47]. The foam is created by rhythmical abdomen movements: the abdominal tip submerges in the fluid, and dipping and rolling motions accompanied by contractions within the fluid force a bubble out of the channel opening and produce several bubbles (approx. one bubble per second) before the air supply need be replenished. The body is fully covered in foam within 15–30 min, and spittlebug nymphs appear to produce foam continuously [31,46,48,49]. Proteins and mucopolysaccharides act as surfactants, stabilizing the foam bubbles [34,37–44]. Interestingly, the foam is inhabited by various tiny creatures, such as amoebae, infusoria, rotifers [31], bacteria [33,35,50] and dipteran larvae [51], which metabolites may also influence the foam properties.

Humidity is a crucial factor in spittlebug life and is required for survival and reliable foam production and quality [48,52–54]. Outside the foam, the smooth, slippery, ‘thin-skinned’ nymph surfaces dry out fast, leading to high mortality [31,55]. The grouping of numerous nymphs, resulting in huge foam nests and stable microclimate, apparently increases their survival [17].

Besides keeping the water balance, various other effects and functions have been attributed to the spittlebug's foam. (i) Antipredatory properties have been reported towards birds [56], spiders and ants by chemical mimicry (via olfactory cues) [55] and physical interactions, e.g. with ants grooming their antennae after contacting the foam [31,57,58]. Prairie ants may even collect spittlebug foam to construct their aphid tents [59]. (ii) Antimicrobial activity has been recently demonstrated by Sahayaraj *et al*. [60]. (iii) The foam's multiple scattering of light in all directions causes the white foam colour [1,61], which might function as a physical signal to herbivorous insects and mammals commonly avoiding such whitish structures in nature already occupied by other phytophagous species [62]. (iv) The foam reflection protects from bright light (1800–2000 lux) and UV radiation. Both cause higher nymph mortality and lower foam production [45] or toxic effects [63,64]. (v) The control of solar input via foam allows bugs' thermoregulation [65], providing a constant thermal microhabitat [58]. (vi) Because sounds in foam run slower and less far, the foam provides mechanical insulation against knocks and shock waves [1,66]. (vii) Foam supports the nymphs' attachment to plant surfaces, preventing the dislodgement from hosts. Leaves reduce runoff and evaporation of spittlebug foam [67].

Considering the diversity of host plants, the foam must be universal, wetting a broad range of plant surfaces having water contact angles from 0° up to 163° [68–70]. One wonders if spittlebug foam behaves similarly to synthetic surfactant solutions on leaf surfaces, which lead to higher wettability, surfactant coverages, plant surface alterations and surfactant-induced biochemical plant responses [71–73]. In this context, surface/solid–foam fluid interactions and foam properties matter.

While the effects mentioned above have been previously experimentally studied, distinct information about foam adhesion is still lacking. Several hints in the literature let us expect foam stickiness because a mucilaginous substance renders it viscous [29], and the tenacious foam fluid can be drawn between the fingers into viscid threads, leaving behind a gummy mass after evaporation [15,17,31]. The foam can stick to a vertical surface owing to its elasticity and ability to cover and cling to surfaces [1]. Further, due to its adhesive properties, ‘cuckoo spittle' traps passing small invertebrates, such as mites, spiders, springtails, thrips, leaf- and planthoppers, flies and butterflies, and it has been observed to glue the mouthparts of ants and spiders and renders them at least temporarily unfunctional [35,74,75]. Thus, we aimed to study foam fluid–solid interactions synergistically, applying microscopic visualization, desiccation and contact angle measurements, and pull-off force tests. In addition, video recordings and behavioural assays with spittlebug nymphs and predators dealing with the foam were carried out. The ecological implications related to the multifunctional foam effects are discussed.

## 2. Material and methods

### 2.1. Insects

For behavioural assays, spittlebugs *Aphrophora alni* Fall. (Hemiptera, Aphrophoridae) were collected from various weeds in a moderately humid grassland and marshes in Hermsdorf (Berlin, Germany; 52°37′ N, 13°19′ E) and kept in the laboratory fed with various host plants, including *Artemisia dracunculus* L. (Asteraceae), *Artemisia* sp., *Ballota nigra* L. (Lamiaceae) and *Bromus* L. sp. (Graminaceae). Spittlebugs in the collection field areas commonly settled on these host plants used for laboratory rearing and studies. In addition, living workers of the black garden ant *Lasius niger* L. (Hymenoptera, Formicidae) and a non-specified spider were collected from a ruderal area in Berlin and *Myrmica rubra* L. (Hymenoptera, Formicidae) wayside in Büsnau (Stuttgart, Germany; 48°44′ N, 9°6′ E). Ants, including *L. niger* and *M. rubra*, as well as spiders, have been previously and frequently observed contacting and being repelled by spittlebug foam [20,35,55,57,59,74]. That is why we have included them in behavioural and microscopic observations.

For contact angle measurements, weighing and adhesion tests, living foam-covered fourth instar nymphs of *A. alni* were collected from various weeds in a moderately humid grassland wayside in Büsnau (Stuttgart, Germany; 48°44′ N, 9°6′ E). They were kept in ventilated plastic boxes (Exoterra faunariums, 360 × 170 × 220 mm^3^ with ventilated lids; HAGEN Deutschland GmbH & Co. KG, Holm, Germany) on turgescent host plants (*Potentilla* L. sp., Rosaceae; *Plantago lanceolata* L., Plantaginaceae) up to the analyses for maximum 40 days under laboratory conditions at 23 ± 2°C, 80 ± 5% relative humidity, and a daily photoperiod of 16 h.

### 2.2. Video recordings

Videos at a magnification of up to ×150 were recorded using the stereomicroscope Olympus SZX12 combined with Olympus DF PLFL 1.6× PF 0.3× and 0.5× objectives, the light source Highlight Olympus Europe 3100 (Olympus Corp., Tokyo, Japan), and the camera Panasonic Convertible AW-E 300 E (Panasonic Broadcast & Digital Systems Company, Division of Matsushita Communication Industrial Co. Ltd, Yokohama, Japan). Video sequences were saved via the super VHS ET video recorder JVC SR-versus 30 (JVCKENWOOD Deutschland GmbH, Bad Vilbel, Germany) on Panasonic AY-DVM63PQ MiniDV tapes (Panasonic Broadcast & Television Systems Company, Division of Matsushita Electric Industrial Co. Ltd, Systems Business Group, Osaka, Japan). Editing was done with Adobe Premiere 6.0 software (Adobe Systems Inc., San José, CA, USA).

### 2.3. Behavioural assays

#### 2.3.1. Foam fluid contacts by black garden ants

Two droplets of tap water and two droplets of foam fluid (each 5–7 mm in diameter, about 150 µl) were distantly placed in a cleaned glass Petri dish of 130 mm diameter, covered with a Plexiglas plate. An ant was released to this arena and observed for 30 min. The behavioural sequences were recorded manually; the number of contacts with different fluids was counted. This procedure was individually repeated with 12 ant workers. Ants established in the arena chose between water and spittlebug foam fluid, corresponding to the situation on partly spittlebug-foam-covered plant surfaces in a humid environment.

#### 2.3.2. Handling of fluid by black garden ants

Glass troughs (‘Blockschale', 40 mm × 40 mm, 15 mm high, spherical trough of 28 mm diameter and 10 mm depth; Aug. Hedinger GmbH & Co. KG, Stuttgart, Germany) were about half-filled (45 ml, approx. 2 mm high level) with either tap water or foam fluid. Thus, the ants did not dive their whole body but submerged with their legs. A single ant worker was released into the fluid, and the times needed to escape and to groom after escape were recorded for 15 min. The observations were carried out separately with eight individual ant workers. The setup resembles an ant entangled with legs in the spittlebug foam after first contact and adhesion.

### 2.4. Microscopy

To observe the gross morphology, a Nikon Coolpix E995 digital camera was used separately and in combination with a stereomicroscope (Olympus SZX12), equipped with a DF PLAPO 1_PF objective (Olympus Corporation, Tokyo, Japan), adapted to the stereomicroscope with a C-Mount adapter and an MDC2 relay lens MXA29005 (Nikon Corporation, Tokyo, Japan).

Microstructural studies were carried out with a cryo-SEM Hitachi S-4800 (Hitachi High-Technologies Corp., Tokyo, Japan) equipped with a Gatan ALTO 2500 cryo-preparation system (Gatan Inc., Abingdon, UK). Fresh samples of foam-covered larvae on and off host plant pieces were mounted on metal holders by polyvinyl alcohol Tissue-Tek OCT Compound (Sakura Finetek Europe BV, Zoeterwoude, The Netherlands). Then, the samples on the holder were frozen in the preparation chamber at −140°C, sputter-coated with gold–palladium (6 nm thickness), and examined in a frozen state in the cryo-SEM at 3 and 5 kV accelerating voltage and −120°C temperature. Also, foam cross sections were visualized after shock-freezing and freeze-fractured foam samples using a cold knife in the preparation chamber. For comparison, several specimens were separately submerged for 5 min in 50 ml Aqua Millipore water to remove the foam. Exemplarily, a *Myrmica rubra* L. (Hymenoptera, Formicidae) worker after contact with the foam was observed.

### 2.5. Contact angle measurements

Glass microscope slides (ISO 8037/I, Carl Roth GmbH & Co. KG, Karlsruhe, Germany) were used as test substrates and cleaned before experiments by successive immersions in piranha solution (mixture of sulfuric acid H_2_SO_4_ and hydrogen peroxide H_2_O_2_, 3:1), rinsed with Aqua Millipore water and dried immediately by means of compressed air. To hydrophobize the glass surface, it was silanized with 1*H*,1*H*,2*H*,2*H*-perfluorodecyltrichlorosilane 97% (C_10_H_4_Cl_3_F_17_Si, SIH5841.0, ABCR GmbH & Co. KG, Karlsruhe, Germany).

Measurable, well-shaped ‘de-aerated' drops without air bubbles were obtained by removing the air, alternately sucking up and dispensing it quickly using glass Pasteur pipettes (long form, total length 230 mm, no. 567/2, Assistent, Glaswarenfabrik Karl Hecht GmbH & Co. KG, Sondheim, Germany).

For comparison, static contact angles of ‘de-aerated' foam fluid and Aqua Millipore water to (a) normal and (b) hydrophobic silanized glass surfaces were measured using the sessile drop method (drop volume 5 µl). Foam fluid drops were manually positioned on the glass surface with glass Pasteur pipettes. Aqua Millipore water was released by the automatic drop dispenser of an OCAH200 high-speed optical contact angle measuring device (Data-Physics Instruments GmbH, Filderstadt, Germany). The ellipse fitting was applied to the obtained droplet images (SCA20 3.7.4 software, Data-Physics Instruments GmbH, Filderstadt, Germany).

Statistical differences of contact angle values (i) between fluids on the same surface and (ii) between surfaces for the same fluid were evaluated using Sigma Stat 3.1.1 software (Systat Software, Inc., Richmond, CA, USA).

### 2.6. Evaporation tests

The mass of ‘de-aerated' foam and Aqua Millipore water on cleaned glass slides (free surface energy: 25.9 mN m^−1^; polar component: 11.9 mN m^−1^; disperse component: 14.0 mN m^−1^; see §2.4 for the substrate preparation method) was continuously recorded with an ultra microbalance (UMX2) and LabX pro balance software (Mettler Toledo GmbH, Greifensee, Switzerland). An initial droplet mass of 3–5 mg was placed on a slide and measured for 3000 s at 5 s intervals.

Ten droplets per fluid were considered at 23.5 ± 1.4°C and 46.4 ± 6.5% relative humidity. Statistical differences between the fluids were elucidated with Sigma Stat 3.1.1 software (Systat Software, Inc., Richmond, CA, USA).

### 2.7. Adhesion tests

Five millilitre droplets of ‘de-aerated’ foam fluid were placed on cleaned glass slides (free surface energy: 25.9 mN m^−1^; polar components: 11.9 mN m^−1^; disperse components: 14.0 mN m^−1^; see §2.4 for the preparation method). A sapphire ball (3 mm diameter, Al_2_O_3_, Product Code AL66-SP-000110, GoodFellow, Cambridge, UK) fixed to a force transducer (10 g capacity, Biopac Systems Ltd, Santa Barbara, CA, USA) combined with a motorized micromanipulator DC3314R and a controller MS314 (World Precision Instruments Inc., Sarasota, FL, USA) was moved up and down at a velocity of 200 µm s^−1^. Thus, it was brought in contact with the foam fluid at an initial load of 6.3 mN and pulled off after (a) 10 min and (b) 30 min. The pull-off force and energy of separation were calculated from obtained force–time curves (AcqKnowledge 3.7.0 software; Biopac Systems Ltd, Goleta, CA, USA).

In total, 71 measurements were performed on the foam fluid and 29 on the clean glass. Mann–Whitney rank sum test was used to statistically compare the forces and adhesion energies (areas under the force curves).

Adhesion tests and contact angle measurements (§2.5) were carried out at 23.0 ± 0.6°C and 48.7 ± 6.5% relative humidity.

## 3. Results

### 3.1. Insect behaviour

**Figure 1.**
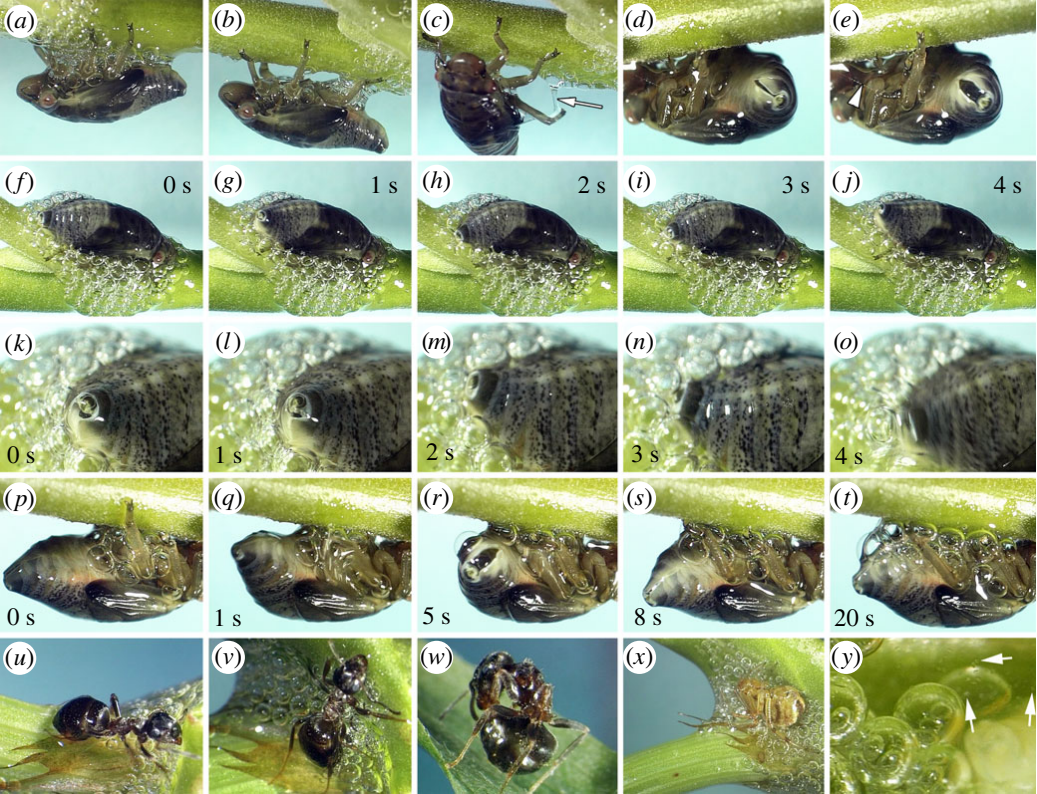
Video stills of *Aphrophora alni* nymphs, predators and commensals in spittlebug foam. (*a*–*c*) A nymph moves in the foam without hindrance, pulling bulks (*b*) and filaments of foam (*c*; arrow). Note the glossy appearance of the body surface, which is totally covered with anal fluid. (*d*,*e*) The anal tip with the ventral channel is initially opening (*d*) and fully opening (*e*). Note the pulled bead-on-string structure of foam fluid in (*e*) (arrow tip). (*f*–*j,k–o*) Sequences of foam bubble release. The abdominal tip raises over the foam (*f*,*g*,*k*,*l*,*m*) to take air into the open ventral channel. Then, the abdominal tip with the closed channel moves into the foam (*h*,*n*) to release a bubble (*i*,*o*) before raising again (*j*). (*p*–*t*) A sequence of foam bubble aggregation, which starts ventrally before covering all the body after about 2–3 min. The ventral tip alternately extends (*p*) and bends (*q*) before releasing a bubble (*r*). The bubbles aggregate (*s*), forming the foam (*t*). (*u*–*w*) An ant trapped in spittlebug foam struggles to free itself (*u*,*v*) and grooms intensively after escaping from foam (*w*). (*x*) A spider entangled in the foam. (*y*) Ciliates belonging to Colpodea move actively in the foam (arrows).

Video recordings elucidate how spittlebug nymphs produce foam bubbles and move without hindrance in the foamy material (figure 1*a–t*; electronic supplementary material, movies S1–S4). Unlikely, predators in contact with the foam struggled to get free and intensively groomed themselves (figure 1*u–x*; electronic supplementary material, movie S5). Bulks, pulled filaments, and bead-on-string structures suggest foam adhesive properties (figure 1*b–e*). Interestingly, ciliates occurred in the foam fluid (figure 1*y*).

**Figure 2.**
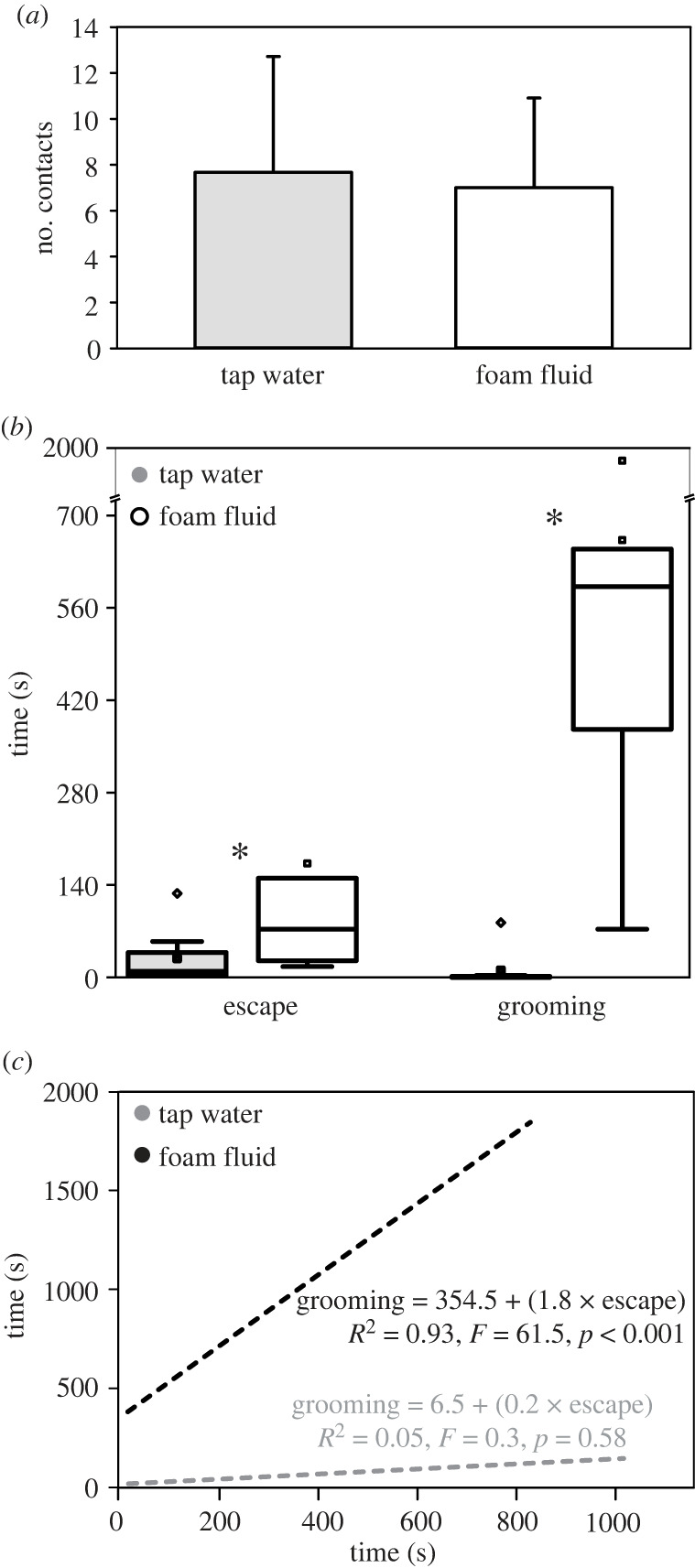
The outcome of behavioural assays with *Lasius niger* ant workers and spittlebug foam. (*a*) The number of contacts with tap water and foam droplets during 30 min in a 13 cm diameter glass Petri dish (*χ*^2^ = 70.0, *p* = 0.3, *n* = 12 per fluid); means and standard deviations. (*b*) The time required to escape and groom after submerging all legs in the spittlebug foam and tap water. Asterisks indicate statistical differences: time to escape, Mann–Whitney rank sum test, *T* = 75.0, *p* = 0.029; time to groom: *T* = 83.0, *p* ≤ 0.001; *n* = 8 per fluid and observation; box-and-whisker diagrams with the ends of the boxes defining the 25th and 75th percentiles, with a line at the median and error bars depicting the 10th and 90th percentiles. (*c*) Relationship between time to escape and groom; linear regressions; regression equations, coefficients, *F* statistics and probability values.

Behavioural assays support video recordings. The ants came in contact with both water (7.7 ± 5.1 times per 30 min; mean ± s.d.) and foam fluid (7.0 ± 3.9 times per 30 min); the number of contacts within 30 min did not differ between tap water and foam fluid (*χ*^2^ = 70.0, *p* = 0.3; figure 2*a*). The initial contact mainly was established between the foam and the head, i.e. the mouthparts and antennae. All contacts lasted not longer than 1–2 s. Ants seemed to avoid contact and did seldomly groom. Some ants turned away from the foam; others paused for a while with sometimes moving mouthparts. A few individuals stepped into the foam with their forelegs. Once partly submerged, having all legs and the body in contact with the foam, ants move intensively to free themselves. At the margin of the glass trough concavity, they tried to pull up with the sliding forelegs, which took considerable time (171.3 ± 293.2 s; significantly more time than in tap water, 27.4 ± 43.6 s; Mann–Whitney rank sum test, *T* = 75.0, *p* = 0.029; figure 2*b*). After escape, ants' bodies were heavily covered with foam. They attempted to slough off the foam fluid on the glass Petri dish surface. Then, they groomed antennae, mouthparts and legs, starting distally, proceeding proximally, and frequently applying the secretion released from the abdominal gland to their integument surface. After grooming, the ants behaved normally without hindrance. They groomed rarely and significantly less after contacting tap water (10.8 ± 29.21 s after water versus 662.6 ± 548.5 s after foam; Mann–Whitney rank sum test, *T* = 83.0, *p* ≤ 0.001; figure 2*b*). Also, the legs were not submerged into the water; due to surface tension, ants could walk over the water surface. Interestingly, the time required for escape from foam is significantly related to the subsequent time of grooming (linear regression, *R*^2^ = 0.93, *F* = 61.5, *p* < 0.001; figure 2*c*).

### 3.2. Foam structure

**Figure 3.**
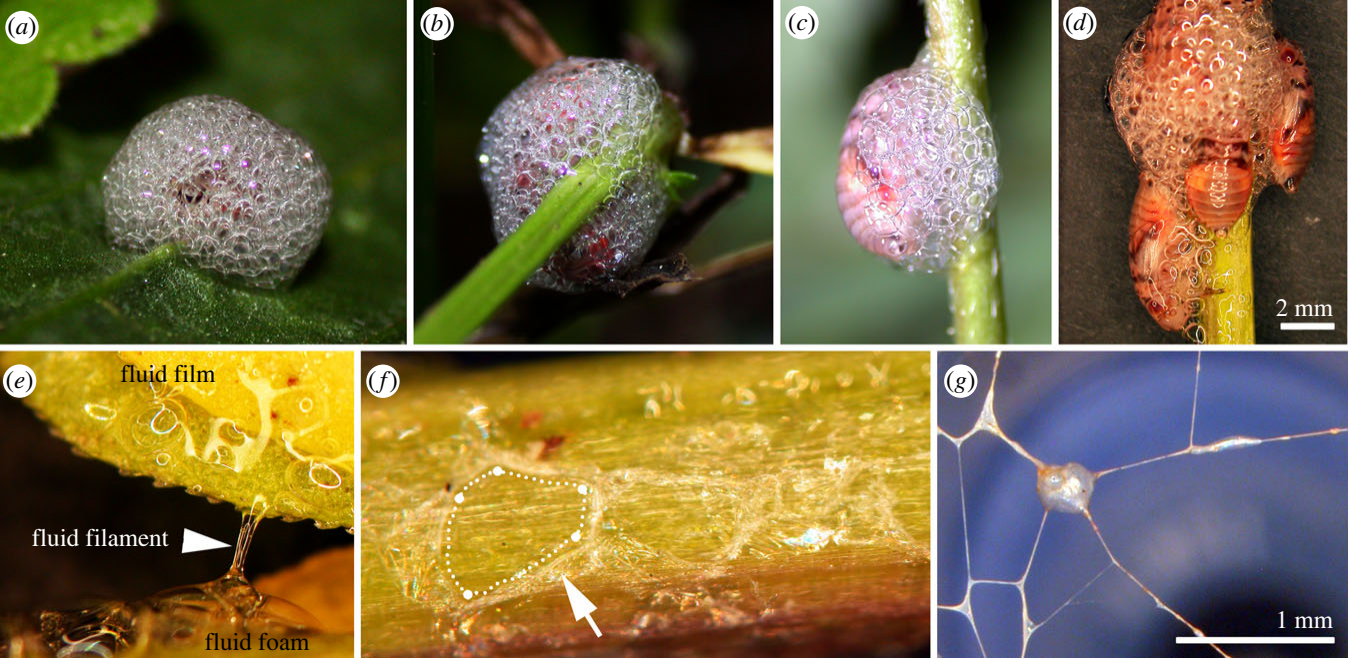
(*a*–*d*) Photographs of *Aphrophora alni* foam nests on the abaxial leaf lamina of ribwort *Plantago lanceolata* (*a*) and cinquefoil *Potentilla* sp. stems (*b*–*d*), occupied with single larva (*a*,*c*) and groups of larvae (*b*,*d*). The foam is built of regular, tightly packed circular and pentagonal bubbles surrounded by fluid lamellae. Note the foam iridescence in (*a*,*b*). (*e*–*g*) Stereomicroscopic images showing different aspects of the foam fluid. (*e*) A fluid film includes few single gas bubbles, fluid foam consisting of larger tightly packed gas bubbles, and a fluid filament pulled between two cinquefoil leaflets. (*f*) Dried foam residues on a cinquefoil stem. Dried lamellae (arrow) form the pentagonal shape established in drying/draining foam bubbles (dotted line). (*g*) Foam dried in a frame, leaving very thin, stable filaments (dried lamellae). The pentagonal bubble shape and nodes between the lamellae of different bubbles are still recognizable.

The spherical to oval wet foam nests appear stable and built of regular bubbles, circular at the foam nest bottom and polyhedral towards the nest tip (figure 3*a–d*). The foam adheres to various plant species, including different surface structures, chemistries and geometries, such as flat leaf laminas (figure 3*a*) and tube-shaped leaf petioles and plant stems (figure 3*b*–*d*). Spittlebug nymphs live individually (figure 3*a*,*c*) or grouped (figure 3*b*,*d*). The foam fluid may be pulled into bulky and thin filaments (figure 3*e*,*g*). After drying out, lamellate and filamentous traces remain on plant surfaces according to pentagonal bubble borders (figure 3*f*). Foam packed and dried within a frame leaves a thin filament pattern that shapes pentagonal bubble borders and converging edges.

**Figure 4.**
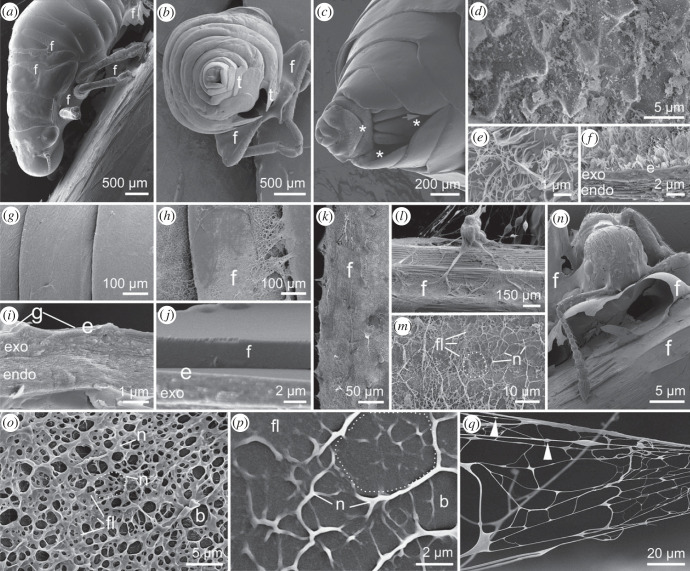
Cryo-SEM images of *Aphrophora alni* third-instar nymphs and foam fluid (residues) on insect and plant surfaces. (*a*) Lateral view of a nymph attached upside down on a *Potentilla* sp. stem. The concavities between body segments are covered with foam fluid. (*b*) Back view of the nymph showing the anal tip and seven proximal tergites with broadened lobes forming a channel. Note the foam fluid trapped between leg segments. (*c*) Close-up of the ventral abdominal tip and anal valves with a matt coverage (asterisks). The arrow points to the opening of the channel formed by tergites' lobes. (*d*–*f*). Surface close-ups of the anal valves and channel (asterisks in *c*): cone-shaped outgrowths covered with protruded thin wax filaments which are partly coalesced; top view (*d*,*e*), cross-section (*f*). (*g*,*h*) Dorsal abdominal nymph surface after washing with water (*g*) and untreated covered with foam, appearing like a meshed network (*h*). (*i*,*j*) Cross sections of abdominal cuticle after washing (*i*) and untreated, covered with foam fluid (*j*). (*k*) A nymph femur covered with a meshed network of foam fluid. (*l*,*m*) A *Potentilla* sp. stem covered with foam fluid pulled into bulk and thin filaments under tension (*l*) and foam bubbles indicated by Plateau borders (dotted line), fluid lamellae and nodes (*m*). (*n*) For example, a *Myrmica rubra* worker entangled in spittlebug foam fluid, embedding the mouthparts and legs. (*o*,*p*) Round and pentagonal bubbles surrounded by fluid lamellae forming the so-called Plateau borders (dotted lines) and converging nodes in a dense foam (*o*) and foam residues on spittlebug nymph cuticle (*p*). (*q*) Under tension, the foam fluid lamellae may be pulled into very thin filaments. Note the bead-on-string structures (arrow tips). Note that the watery foam content is evaporated in all SEM micrographs. b, bubble; e, epicuticle; endo, endocuticle; exo, exocuticle; f, foam fluid; fl, fluid lamella; n, nodes between fluid lamellae of different bubbles; g, epicuticular grease; t, tergites' lobes.

At higher resolution, the foam spreading and wetting are visible on insect and plant surfaces (figure 4). The foam fluid merges in body concavities and ventrally on nymphs taken out of foam (figure 4*a*,*b*). The ventral abdominal tip and the channel inside built of proximal tergites with broadened lobes are structured with micrometre-sized, cone-shaped outgrowths and densely covered with partly coalesced wax filaments of about 2 µm length and 30 nm width (figure 4*c*–*f*). The dorsal integument surface appears smooth after washing and removing the foam with water (figure 4*g*,*i*), while non-treated samples were well wetted with foam fluid, visible as thick top film in cross sections (figure 4*h*,*j*). The nymph dorsal abdominal integument is about 4 µm thick, consisting of a 3 µm thick endocuticle and 1 µm thick exocuticle, which is covered by a *ca* 100 nm thick greasy layer. The foam coverage on all spittlebug nymph body parts and plant surfaces resembles dense meshed networks (figure 4*k*–*m*). The foam fluid is proven to fully cover predators' mouthparts, such as those of *Myrmica rubra* worker ants, after contact with foam nests (figure 4*n*). Details of the froghoppers' spittle match ideal foam properties. Foam fluid lamellae (so-called Plateau borders), nodes (vertexes) between fluid lamellae of different bubbles, the typical spherical shape of liquid foam bubbles, and the pentagonal shape of older or drying foam bubbles are clearly visible (figure 4*o*–*q*). Bead-on-string structures occur on thin, pulled filaments (figure 4*q*).

### 3.3. Foam fluid wetting properties

**Figure 5.**
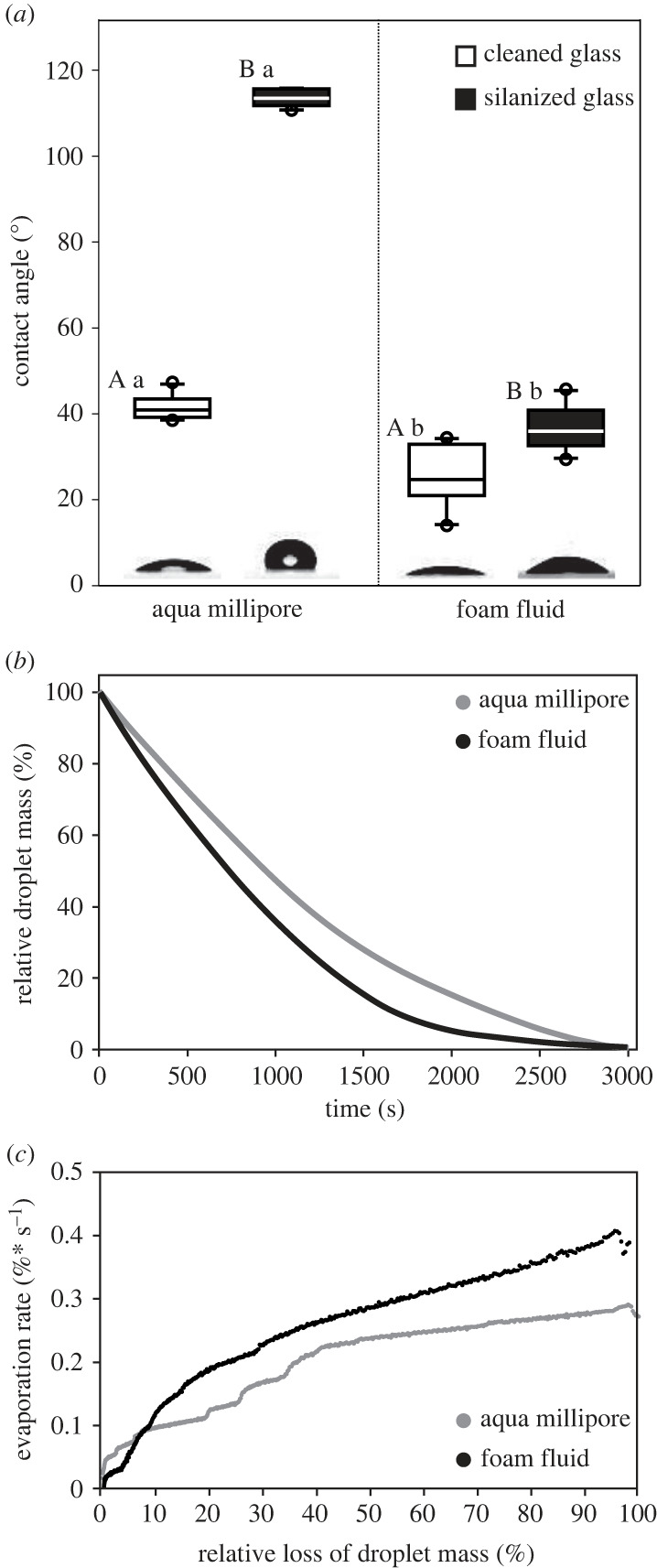
(*a*) Contact angles of Aqua Millipore water and spittlebug foam fluid. Box-and-whisker diagrams: the ends of the boxes define the 25th and 75th percentiles, with a line at the median and error bars depicting the 10th and 90th percentiles. Difference between surfaces for foam fluid: *t*-test, *t* = −4.1, *p* ≤ 0.001 (uppercase letters). Difference between surfaces for Aqua Millipore water: *t*-test, *t* = −66.0, *p* ≤ 0.001 (uppercase letters). Difference between fluids on hydrophobic (silanized) and hydrophilic (cleaned) glass for each substrate: Mann–Whitney rank sum test, *T* = 155.0, *p* ≤ 0.001 (lowercase letters); *n* = 10 per fluid and substrate. The insets show droplet images obtained during contact angle measurements. (*b*) Relative loss of fluid drop mass due to evaporation; ‘means and standard deviations per 5th second'. (*c*) Temporary evaporation rate [5 s × ((Mn − Mn + 1)/5)] obtained from relative values. See table 1 for the half-life period (*t*_50_) and time point of evaporation of 80% (*t*_80_) of Aqua Millipore and cicada foam fluid.

Compared to Aqua Millipore water (contact angle on normal glass: 41.6° ± 2.85°, on silanized/hydrophobic glass: 113.41° ± 1.93°; mean ± s.d., *n* = 10), the ‘de-aerated’ spittlebug foam fluid significantly wetted stronger (Mann–Whitney rank sum test, *T* = 155.0, *p* ≤ 0.001; figure 5*a*). However, the contact angle of foam fluid with normal glass (25.6° ± 7.14°) was lower than with silanized one (36.9° ± 5.16°) (*t*-test, *t* = −4.1, *p* ≤ 0.001).

**Table 1.** Half-life period (*t*_50_) and time point of evaporation of 80% (*t*_80_) of Aqua Millipore and cicada foam fluid (mean ± s.d.) as well as *t-*test parameters (*t*, *t*-statistics; *p*, probability value; *n* = 10 per fluid).

parameter	Aqua Millipore	foam fluid	statistics
*t*_50_ [t(0,5 M0)], s	1026.0 ± 268.55	827.5 ± 199.89	*t* = −1.875; *p* = 0.08
*t*_80_ [t(0,2 M0)], s	1782.0 ± 472.15	1488.0 ± 350.52	*t* = −1.581; *p* = 0.13

### 3.4. Foam fluid desiccation

Though both liquids' masses tended towards zero about 50 min after starting the weighing measurements, the ‘de-aerated’ foam fluid evaporated faster than Aqua Millipore water (figure 5*b*,*c* and table 1). This difference was significant during the first half of the experiment (table 1, *t*_50_), as indicated by the stiffer slope for foam fluid mass loss in figure 5*b* and the higher evaporation rate in figure 5*c*. Interestingly, the foam evaporation rate was lower for the first 10% droplet mass loss than water.

### 3.5. Foam fluid adhesion

**Figure 6.**
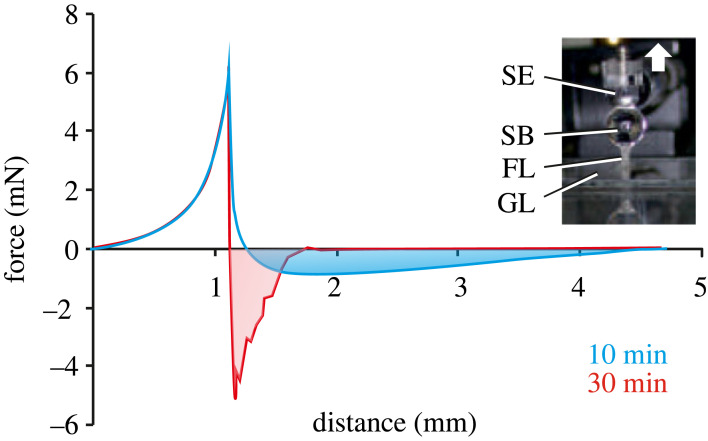
Adhesive properties of the foam. Force–distance diagrams obtained between the clean glass surface and the sapphire ball in the presence of the foam under an initial load of 6.3 mN. The blue curve was obtained after 10 min of foam preparation. The red curve was obtained after 30 min of foam preparation. The area under the negative part of the curve corresponds to the energy of the separation. The inset illustrates the experimental design. The arrow indicates the direction of the pull-off. FL, foam filament; GL, glass surface; SB, sapphire ball; SE, force sensor.

**Figure 7.**
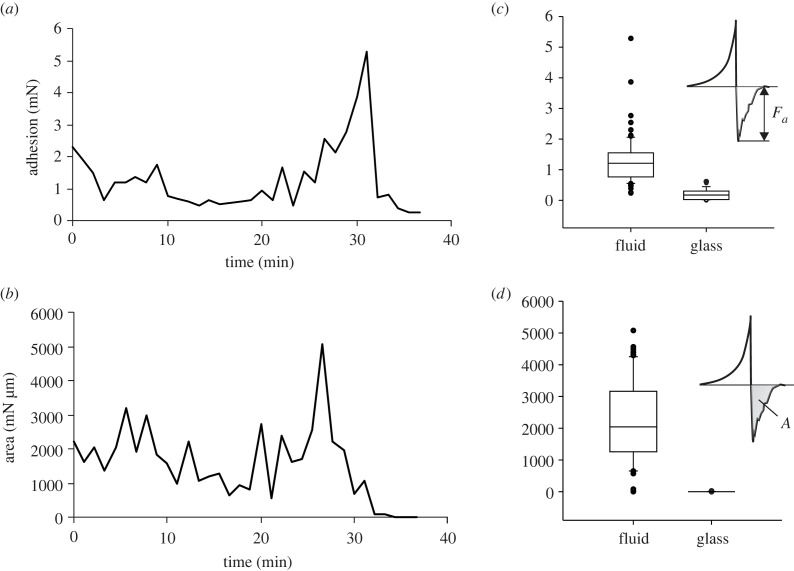
Adhesive properties of the foam fluid. Data are based on the force–distance diagrams (figure 1). (*a*,*b*) Pull-off force *F_a_* (*a*) and area under the pull-off curve *A* (separation energy) (*b*) measured at an individual sample over time (*n* = 36 single measurements). (*c*,*d*) Box-and-whisker diagrams of the pull-off force (*c*) and area under the pull-off curve (separation energy) (*d*) of the fluid compared to the control experiments (dry sapphire on glass). The ends of the boxes define the 25th and 75th percentiles, with a line at the median and error bars representing the 10th and 90th percentiles. A statistically significant difference exists between data obtained on the fluid and the control experiment (Mann–Whitney rank sum test; force: *T* = 2027.0, *p* ≤ 0.001; area: *T* = 2053.0, *p* ≤ 0.001).

The maximum pull-off force and separation energy were measured 25–40 min after foam sample preparation (figures 6 and 7). The effect is due to evaporation and concentration increase of the fluid. Foam fluid adhesion and required separation energy were significantly greater than those of Aqua Millipore water on normal glass (figure 7*c*,*d*). The pull-off force depended on foam fluid hydration status and is higher the more drained the fluid (figure 7*a*,*b*). However, after 25–27 min of dehydration, the fluid reaches maximum adhesion and afterwards rapidly loses its adhesive properties due to drying out. Interestingly, there is some discrepancy between the peaks for the maximum force and maximum separation energy with a certain delay (5–7 min) of the maximum force peak (figure 7*a*,*b*).

## 4. Discussion

### 4.1. Spittlebug nymphs in foam nests

Spittlebug foam surrounds and protects nymphs in different ways (e.g. [1,31,35,55,67]). The present data confirm that nymphs are fragile because of their smooth, shiny, considerably thinner and almost transparent integument [17,45]. Their dorsal abdominal 4 µm thick cuticle resembles that of blowfly legs [76] but differs much from the 35–50 µm thick locust sternal cuticle [77] and 65 µm thick cuticle of dung beetle elytra [78]. The thickness of epicuticular grease (31 nm) ranges in that of blowfly legs (20 nm) [76] but is thicker than that of Colorado potato beetle elytra (8 nm) [79].

The wax filament coverage on the ventral abdomen and inner ventral channel fits the earlier reported presence of wax plates released from the Batelli glands, which produce a lipoid substance [80], causing a milky appearance [17]. The proven hierarchical arrangement of cone-shaped outgrowths and wax filaments indicates anti-adhesive, superhydrophobic, air-retaining properties. This functional surface supports the hypothesis that gas exchange in spittlebug nymphs is similar to that found in plastron-breathing aquatic insects, which retain a bubble of air attached to a layer of hydrophobic hairs [81].

As demonstrated by video recordings and bioassays, spittlebug nymphs in the foam are well protected from predators, hampered by the foam (figures 1*u*–*x* and 2). The amphiphilic foam covers predators' bodies. Surfactant secretions released by insects, such as oral secretions of *Spodoptera exigua* Huebner caterpillars (Lepidoptera, Noctuidae), have been previously reported to defend against enemies by stopping and engaging them in extensive cleaning [82]. Trapped ants invested significant time and effort to escape the viscous spittlebug foam and groom, cleaning the body from extensively adhering foam. ‘Unscheduled' grooming is energy-consuming [83], impacting insects' fitness.

Knowing the importance of free water uptake in ants' water balance [84], one may assume that water droplets attract them. This tendency may result in fatal consequences if attracted to water-similar but sticky foam, which was visited at a comparable frequency to water (figure 2*a*).

### 4.2. Foam structure and dynamics

The visualized foam in contact with insect and plant substrates resembles, for example, the watery–sugary–proteinaceous sundew tentacle secretion [85] and a sugar solution on waxy *Asparagus officinalis* needle leaves [86] or synthetic surfactants [87–89]. The wetting on normal (contact angle 26°) and silanized glass (contact angle 37°) suggests the foam fluid's amphiphilic properties, having probably slightly higher polar and lower disperse portions. Thus, the foam spreads over various surfaces, mainly including hydrophobic, lipophilic plant and arthropod cuticles [68,90], and establishes stable meshworks consisting of fundamental components of the foam [1,3,91]: bubbles, fluid lamellae (Plateau borders) and nodes (vortexes) between three fluid lamellae of different bubbles. The coexisting sugars can stabilize proteinaceous structures [44,92]. The foam appears as wet foam shortly after release, blowing air into the fluid. At this stage, the bubbles are spherically shaped and densely packed. Such a foam comprises many interfaces and, thus, an enormous surface area per unit volume. Knowing that 50 or 100 g of water allow producing a litre of foam with 10 m^2^ of an interface [1], spittlebugs use an effective material available in large volumes to protect themselves. The grouping of nymphs even leads to much larger foam amounts, increasing their survival [17].

Upon ageing, the foam fluid drains (e.g. due to gravity or desiccation), the liquid content reduces, and the bubble shape turns pentagonal. Dried foam leaves lamellate and filamentous, obviously protein and mucopolysaccharide residues on plant and insect surfaces or between frames, suggesting similar substrate surface alteration as known by synthetic surfactants [71,73].

Interestingly, the present study revealed faster evaporation of *Aphrophora alni* spittlebug foam fluid than water, which contradicts previous studies with *Aphrophora saratoga* spittlebug foam [81]. The water evaporation from the foam of *Aphrophora saratoga* was lower than from the free-water surface, and a little net reduction of evaporative water loss happened by spittle [81]. This difference could be explained by (i) differences in foam contents between species [4], (ii) the different consistencies of foam (‘de-aerated’ in the present study but bubbly foamed in [81]) and (iii) different temperature and humidity conditions, which are unfortunately not mentioned by [81]. Colder temperatures are known to lead to a higher dry matter content of the foam, and the content of sugar units building the carbohydrate polymer increases significantly [44]. Foam lifetime depends strongly on humidity [93]. However, foam fluid evaporation should not be debilitating to the continuously foam-producing nymphs [49]. Foam desiccation could even be advantageous in preventing predators from gluing their mouthparts or other body parts because the adhesion of drying foam increases, as discussed in the paragraph below.

Although we did not record the contact line while drying de-aerated foam droplets, we suggest less receding of foam fluid droplets than pure water droplets due to the contained molecular networks. The available observations and images of dried (not de-aerated) natural foam (figure 3*f*,*g*; also the foam cryo-SEM micrographs in figure 4 where water evaporated) underpin this consideration: while water evaporated, the foam fluid lamellae and filaments remained and shaped the original droplet area on the substrate. However, the droplet vertex seemed to collapse, which will be proven by future approaches.

### 4.3. Foam fluid stickiness

The pull-off force of foam on normal glass was about five times higher compared to tests 30 min to 10 min after sample preparation (figure 6). The increase in adhesive ability of the foam is thus strongly related to an increased concentration of solid components due to the water evaporation. Solid filaments and lamellae meshwork should lead to considerable load distribution, i.e. forces spreading out over a length, area, or volume, being resistant against external impacts, such as wind, water or earth pushing on a surface.

Since water evaporation is relatively fast (in this study, it is as fast as pure water), especially taking into account small amounts of fluid that can be taken by potential predators, such as ants, wasps etc., the desiccation effect of the fluid can turn it to an effective adhesive, which can contaminate and stick together the surfaces, mouthparts and locomotory systems of predators. For comparison, the traction force of walking and pulling *Myrmica rubra* L. workers (Hymenoptera, Formicidae) on glass and rough polishing paper (P60) is 3.5 mN (D. Voigt 2009, unpublished data). Thus, the drying foam fluid on the predator's surface may stick stronger, effectively protecting spittlebugs in their foamy envelope.

Interestingly, the foam fluid can be pulled into bulky and thin filaments under tension. Occasionally, beads-on-a-string can be observed (figures 1*e* and 4*q*). Such structures and thinning filaments are particularly formed by viscoelastic inertial fluids [94] and are also known from sticky prey-capturing water-based sugary–proteinaceous fluids, e.g. of *Drosera* plants, onychophoran slime or spider webs [95–98].

Wet, bubbly foams like solid bodies (low shear) exhibit elastic properties. They flow and deform similarly to liquids (high shear). Elastic energy is needed to shear the foam network [91]. The character of spittlebug foam fluid's adhesion curve over desiccation time resembles those previously obtained for plant diaspore mucilages, which also have protective and anchoring functions [99–103]. Interestingly, mucilaginous substances of plant diaspores have very low friction at high hydration levels. With an increasing desiccation time and increasing adhesion, friction also increases. Low friction at highly hydrated conditions can also be assumed for the spittlebug foams: a hypothesis which can be tested in future experiments. A further research question in this context is the significance of crosslinking inorganic ions/salts [34,104] and excretions of miniature commensals, such as ciliates (figure 1*y*), in foam fluid adhesion and friction.

## 5. Conclusion

**Figure 8.**
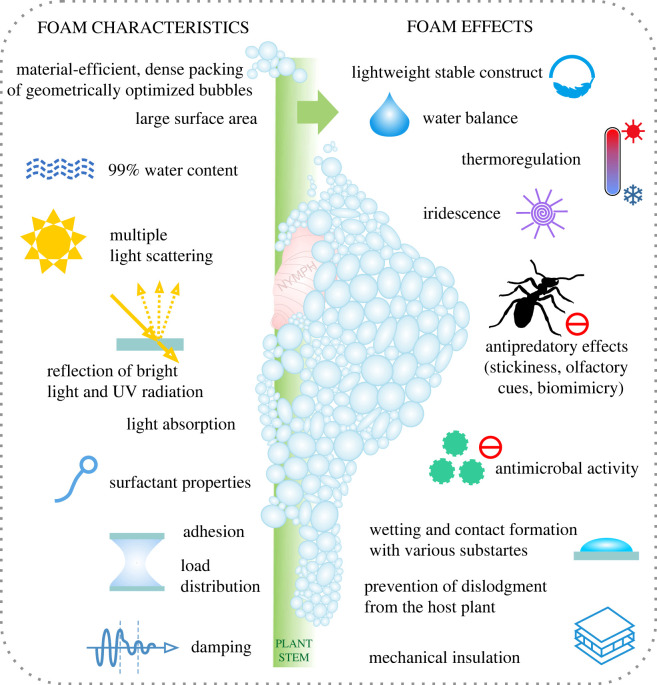
Spittlebug foam characteristics and facilitated integrative effects support nymph protection and survival in multiple ways (schematic chart). The present study is set in context with previous outcomes [1,15,17,29,31,33–35,37–45,48,52–75]. The smart multifunctional foam material may inspire innovative biomimetic and biomedical approaches.

The present study experimentally proves foam adhesion depending on the foam hydration state; the more dry, the stronger adhering to substrates and impacting spittlebug predators. Thus, adhesion is one of the numerous previously reported foam characteristics resulting in multifunctional effects, which promote spittlebugs' survival, including defence against predators, and render the foam a smart, biocompatible material of biomimetic and biomedical interest (figure 8).

The sustainable ‘reuse' of large amounts of excrement for foam production suggests energetic advantages, also by investing in a thin nymph cuticle protected with the foam. Such a strategy should be evolutionarily successful in humid habitats, as confirmed by the independent evolution of foam nests in different groups of organisms, such as spittlebugs, frogs and fishes.

## Data Availability

Data are reproducibly presented in display items and the electronic supplementary material table. Supplementary material is available online [105].
